# Malleable Morality: Re-Shaping Moral Judgments in Health Policymaking

**DOI:** 10.1017/jme.2023.70

**Published:** 2023

**Authors:** Shelly Simana

**Affiliations:** 1: STANFORD LAW SCHOOL, STANFORD, CA, USA

**Keywords:** Health, Moral Experts, Moral Intuition, Moral Judgment, Policymaking, Social Intuitionist Model

## Abstract

When confronted with moral dilemmas related to health, governments frequently turn to “moral experts,” such as bioethicists and moral philosophers, for guidance and advice. They commonly assume that these experts’ moral judgments are primarily a product of deliberate reasoning. The article challenges this assumption, arguing that experts’ moral judgments may instead be primarily a product of moral intuitions which, often subconsciously, respond to the social setting.

## Introduction

In contrast to the increasing skepticism and distrust of experts by a significant portion of the public,^1^ experts are generally well-trusted by governments to act in the public’s interests.^2^ Governments hold that relying on a group of experts with the appropriate expertise and knowledge^3^ is necessary to make policy decisions.^4^ They consider expertise a trustworthy source of credibility that is “synonymous with truth.”^5^

In this article, I focus on a particular type of experts — “moral experts.” Such experts have specialized knowledge and understanding of moral philosophy and ethics (e.g., public health ethics, clinical ethics, and research ethics), and are frequently called upon to advise governments on health-related moral dilemmas.^6^ The involvement of moral experts in health policymaking can take many forms: these include, inter alia, holding positions in public service, participating in advisory committees, organizing policy forums, and publishing reports with the goal of influencing policy in a particular health domain.

It should be stressed that the article’s arguments are not confined to moral experts; rather, they could also apply to other experts whose decisions involve making moral judgments. Consider the design of machine learning-based solutions for health services as an example. When creating and building AI systems, designers and developers implement a set of moral values that act as decision guides. As a result, they make moral judgments in the course of their work.

This article questions the apparent causality of reasoning in national and international health policymaking, addressing the gap between how moral experts *ought* to make and how they do *in fact* make moral judgments on moral dilemmas related to health, health care, and public health. By “moral judgments,” I mean judgments that involve normative assertions (e.g., “mask mandates are justified”), as opposed to descriptive or factual assertions (e.g., “The R0 of Covid-19 is between 5-7”). For this purpose, I employ two observations from the Social Intuitionist Model (SIM) of moral reasoning, which was developed by the social psychologist Jonathan Haidt.^7^ These observations shed new light on the role of moral experts in health policymaking, particularly in areas that raise complex moral issues. I argue that these two observations suggest that it should not be up to moral experts alone to address moral issues of this nature and that cooperation at both the national and international levels is essential.

In this article, I focus on a particular type of experts — “moral experts.” Such experts have specialized knowledge and understanding of ethics (e.g., public health ethics, clinical ethics, and research ethics) and moral philosophy and are frequently called upon to advise governments on health-related moral dilemmas. The involvement of moral experts in health policymaking can take many forms: these include, inter alia, holding positions in public service, participating in advisory committees, organizing policy forums, and publishing reports with the goal of influencing policy in a particular health domain.

The first observation relates to the process by which individuals form moral judgments. According to Haidt, moral reasoning is a “post hoc invention meant to rationalize spontaneous moral intuitions.”^8^ Meaning, intuitions come first, followed by strategic reasoning. When individuals engage in moral reasoning, it is usually after an instinctive process led them to a particular judgment. Interestingly, individuals may not even be aware of the moral intuitions that guide them and may be even less aware of their origin.^9^ Haidt further suggests that one of the only reasons for engaging in moral reasoning is to better prepare for social situations in which individuals may be required to justify their judgments to others.

This observation can be placed within a vast body of philosophy and moral psychology literature that emphasizes the importance of emotions and intuitions. This literature first appeared in Adam Smith’s and David Hume’s writings and was then stressed by psychologists like Freud who assumed that judgments are “driven by unconscious motives and feelings, which are then rationalized with publicly acceptable reasons.”^10^

Haidt’s first observation implies, I argue, that even expertise in ethics and moral philosophy — which is the kind of expertise we expect moral experts will bring to the table — does not necessarily guarantee moral judgments that are largely based on reason and less on moral intuitions. This observation suggests that experts’ moral judgment may be subjective and devoid of deliberate reasoning and reflection; it may ultimately reflect experts’ personal moral intuitions and be guided by an intuitive response. For this reason, I believe that we should start questioning the status of moral experts as reasoned “consultants” and ensure that health policy remains accountable to the interests and needs of the public.

Haidt’s second observation concerns the malleability of one’s moral intuitions, and hence moral judgments. He explains that moral reasoning is not always self-constructed and is mostly received from the outside.^11^ In other words, moral reasoning occurs in a social setting, where individuals can challenge each other’s moral judgments and generate new intuitions.^12^ Haidt maintains that these new intuitions are more likely to result in nuanced and multi-faceted moral judgments because venturing outside of one’s own “moral matrix” helps to develop moral humility and overcome one’s sense of self-righteousness.^13^

I claim that the second observation uniquely demonstrates the value of public engagement and participation and paves the way for a more democratic approach to health policymaking. Specifically, this observation implies that experts’ moral judgment is malleable and may be more reasoned if experts discussed the mitigating factors involved in moral dilemmas with multiple stakeholders, such as individuals representing different backgrounds, cultures, and fields of study. Since it may be challenging — if not impossible — to deny experts’ initial moral intuitions, vigorous public engagement with experts’ moral judgments has the potential to provide a basis for both a well-functioning health system and a just society.^14^

Public engagement and participation promise to restore rationalism in health policymaking by upsetting experts’ initial moral judgments, introducing them to new moral intuitions, and using their reasoning capability to adjudicate conflicts between competing intuitive moral judgments. With a refined moral judgment, moral experts would be able to provide more reasoned recommendations. Moreover, public engagement performs a democratic function by lending legitimacy to health policy decisions that may otherwise be based solely on the normative judgments of experts and other non-elected officials and agencies.

To summarize, in this article, I suggest that experts’ moral judgments may be intuitive, and that the reasons they offer to justify those judgments are likely to be post hoc rationalizations. Consequently, moral experts may make suboptimal decisions from the public’s perspective.^15^ Moreover, I contend that health policy should not be developed exclusively based on the moral judgments of a selected group of experts because these judgments may not always be reasoned. Moral experts can benefit from having their moral judgments questioned through open discussion and debate with members of the public and other stakeholders who may have different moral intuitions and judgments. Ultimately, by opening experts’ moral judgments for evaluation, it would be possible to engage with and consider the judgments of non-experts, promoting more democratic decision-making processes. Overall, I call for a broad public dialogue over health-related moral issues, challenging the prevailing notion that moral reasoning is “beyond the competence of untrained minds” and stressing the importance of not conducting moral deliberations “by expert bodies behind closed doors, with little or no accountability to wider audiences.”^16^

The article is constructed as follows. In Part I, I discuss the role of moral experts in health policymaking. I show that experts have been given a dominant role in health policymaking processes, and that the policy recommendations they provide are often not contested. I also address challenges that arise from placing a greater reliance on the moral judgments of experts.

In Part II, I discuss how SIM can be used in the context of moral experts. I begin by explaining that moral experts, like everyone else, may be motivated by their moral intuitions. Upon receiving a request for policy recommendation, moral experts may develop moral judgment instinctively, and their moral intuition would guide this judgment. I then explain the malleability of experts’ morality. Drawing from SIM, I argue that experts’ moral intuitions can be open to influence from a wide range of stimuli emanating from the social milieu in which they are embedded. This suggests that experts’ moral intuitions and, by extension, their moral judgments on moral issues might shift if they were exposed to individuals from various backgrounds and points of view. Overall, I propose a different lens through which we could challenge rationalism when it comes to moral dilemmas related to health and advance an argument for public engagement in health policymaking.

In Part III, I demonstrate that international cooperation on issues that have global effects is vital. I use a case study on the governance of gene-editing technologies to highlight SIM’s practical implications. I argue that the governance of these technologies is currently dominated by experts and propose other governance alternatives that are both more democratic and just.

## Clarifications

Before going any further, four points of clarification are appropriate. First, SIM is not concerned with the moral judgments of experts; instead, it investigates how moral judgments are made by humans more broadly. In this article, I draw on SIM’s insights and expand them to the realm of moral experts in health policymaking.

One might argue that some moral experts, such as philosophers, may be better equipped to engage their reasoning. Indeed, Haidt himself notes that “a person could, in principle, simply reason her way to a judgment that contradicts her initial intuition,”^17^ and that “the fact that there are at least a few people among us who can reach such conclusions on their own and then argue for them eloquently … means that pure moral reasoning can play a causal role in the moral life of a society.”^18^ While this is true, the ability of certain moral experts to engage their reasoning does not imply that *all* moral experts can do so or that moral experts *always* reason well.

Second, Haidt is not the first to distinguish between intuitive and deliberative judgments. Economics Nobel Prize winner Daniel Kahneman, for example, has famously shown that judgments can be processed by one of two systems: System I, which operates quickly and intuitively, and System II, which operates slowly and thoughtfully.^19^ The primary reason I rely on Haidt in this article is that he focuses on *moral* judgment as opposed to *evaluative* judgment (e.g., probability or quantity assessments).

Third, to the best of my knowledge, SIM has not yet been tested empirically; thus, there is no evidence to prove the validity of the model as a whole. However, as I describe below, SIM’s key assumptions have been studied extensively and are supported by evidence. Hence, it still serves as a valuable model to explain how malleable experts’ moral intuitions and judgments are and how profound the influence of culture and social environments on the formation of those intuitions and judgments is. Let me to briefly describe some of the pertinent evidence that supports SIM’s assumptions.

To begin, various studies have indicated that emotions and intuitions play a substantial role in moral judgments, revealing the limitations of rationalism.^20^ These studies found that moral judgments are primarily motivated and driven by automatic and intuitive processes in the human brain and are difficult to control. To illustrate, one of Haidt’s famous examples is people’s reactions when asked about incest. Haidt and his colleagues observed that most people instinctively say that it is wrong for siblings to have sex, and that only after this immediate reaction do they start looking for reasons why incest is wrong.^21^ These findings are consistent with other studies in which participants were asked whether they would consider eating a dead pet dog, flushing the toilet with the national flag, or eating a chicken carcass that had recently been used for masturbating.^22^ Similarly, Jonathan Baron, a Professor Emeritus of Psychology at the University of Pennsylvania, demonstrated that individuals and government officials in matters like drugs, vaccines, abortion, and birth control “follow intuitive principles of decision making that are not designed to produce the best consequences in all cases.”^23^

In addition, various researchers have long studied the intuitive judgments of (non-moral) experts. One might claim that experts rely less on intuitions than the average person, but evidence — admittedly from outside the moral domain — suggests otherwise. Studies, some of which form the basis of SIM, indicated that experts are no different from other individuals because subjective intuitions and biases also influence their judgments.^24^ For example, Daniel Kahneman, Olivier Sibony (a Professor of Strategy at HEC Paris), and Cass Sunstein (a Professor of Law at Harvard University), found that when individuals — including experts — exercise judgments, they integrate various pieces of information into an overall evaluation of the issue at hand. Each of those judgments involves “noise” due to differences in expertise, personality, and preferences.^25^

Furthermore, it is well-established that social forces, such as persuasion and social environments are fundamental to judgment formatiom.^26^ Different studies, some of which Haidt himself highlights, revealed that judgments can be impacted by others’ judgments and that under certain conditions, new intuitions can be triggered. One study, for instance, found that affective persuasion could change affect-based attitudes.^27^ Another study showed that judges retain ideologies they learned from colleagues with whom they were randomly assigned to serve on the bench. It was discovered that when judges sit with other judges who have previously received economics training and advocate for harsh sentences, they begin to impose harsher sentences themselves.^28^

The fourth and final clarification I would like to make is that I have no intention to denigrate experts, devalue their expertise, or discount their relevance. I am not “anti-expertise.” Moral experts play a crucial role in health policymaking and there is much to learn and gain from their knowledge and experience. I only wish to highlight that moral experts often use their expertise to justify normative claims, and thus, their moral judgment should not be taken as a factual assertion (i.e., an assertion that does not have a normative element in it).^29^ Other stakeholders should participate in formulating health policy *in addition* to moral experts. When making decisions about health, numerous stakeholders should participate and the types of knowledge and experience used should be diverse.^30^

## Part I: The Role of Moral Experts in Health Policymaking

When making decisions on various aspects of individuals’ health, governments often rely on the moral judgment of experts.^31^ Moral experts are consulted on diverse issues,^32^ including complex moral quandaries related to vaccinations, end-of-life care, abortion, and resource allocation.

Consider, for example, the coronavirus pandemic. During the pandemic, different types of experts were instrumental in developing strategies to stop the spread of COVID-19. Various restrictions on people’s freedom, such as the prohibition of large gatherings and the shutting down of businesses, were justified by referring to the epistemic authority attributed to experts.^33^ Governments frequently sought moral experts’ advice and relied on them to solve important problems, especially those pertaining to priority access to vaccines and intensive care unit triage decisions.^34^

Moral experts are granted special status when designing policy because of their (allegedly) reasoned judgment, as well as the expectation that they act for the public’s benefit. It is thought that they possess the highest level of rationality by virtue of their specialized training and moral expertise.^35^ This is why moral experts’ advice is seen as a crucial component of what is often perceived as “rational” policymaking.

This model of “expert governance” imagines a governing system that it is reasoned and free of psychological proclivities^36^ and depends on a set of impersonal, exogenous principles (e.g., cost-effectiveness) that reflect the common good. It is because they purport to reflect no one’s point of view that such principles provide “a view from nowhere,” in the words of Sheila Jasanoff, a Professor of Science and Technology Studies at Harvard University, who has extensively written on the role of experts in science policy.^37^

What is wrong with governments’ increasing reliance on moral experts in health policymaking? First, governments do not recognize that moral intuitions play a significant role in the moral judgments of experts, nor do they acknowledge that experts’ personal motivations could influence their moral intuitions. Expert governance creates an illusion of objectivity and rationalism in terms of how experts arrive at moral judgments.

Second, the growing reliance on moral experts discourages public participation and runs counter to the ideals of a democratic society in which members of the public have an active voice in policymaking. Because following one’s intuitions and biases may result in suboptimal outcomes, and because the public cannot fully participate in decision-making processes, expert governance may fail to represent the interests and needs of the public. It should be clarified that I am well aware that not all members of the public can participate in every decision-making process. The argument I advance is that a growing reliance on moral experts decreases the public’s opportunity and motivation to participate in decision-making processes.

The need to allocate resources during the COVID-19 pandemic exemplifies the above-mentioned concerns. During the recent pandemic, several states in the United States adopted criteria for accessing life-saving ventilators, among them the exclusion of mentally disabled individuals or people with specific pathologies.^38^ The criteria were adopted on the advice of various experts who believed such criteria were “appropriate” under the current circumstances. While they were deemed appropriate, these highly controversial criteria ultimately reflect the “cultural, ideological, political, or religious views or biases” of a small number of experts.^39^

## Part II: Re-Shaping Moral Judgments: Insights from Haidt’s Social Intuitionist Model

While much has already been written about the role moral experts play in policymaking and their impact on public policies, to the best of my knowledge, the origin and development of their moral judgment, particularly how it is shaped, have not yet been explored.

Historically, the Rationalist school of thought in moral psychology held sway for decades as the most widely accepted view.^40^ In a nutshell, rationalists have maintained that moral judgment is primarily motivated by deliberate reasoning.^41^ In practice, this means that before making any moral judgment, individuals analyze and assess a variety of factors, including harm, rights, justice, and fairness, as well as other considerations.^42^

The rationalist approach has been called into question by different researchers, among them Jonathan Haidt.^43^ Haidt developed a comprehensive model, SIM, that was initially presented in his paper “The Emotional Dog and Its Rational Tail.”^44^ SIM, I claim, opens the “expertise black box” and sheds new light on how moral experts may arrive at their moral judgments.

It will be helpful at this point to provide the definitions of the concepts that form the foundation of SIM. *Moral intuitions* are defined as “sudden appearance[s] in consciousness of a moral judgment, including an affective valence (good-bad, like-dislike), without any conscious awareness of having gone through steps of searching, weighing evidence, or inferring a conclusion.”^45^ *Moral judgments* are defined as “evaluations (good vs. bad) of the actions or character of a person that are made with respect to a set of virtues held to be obligatory by a culture or subculture.”^46^ And lastly, *moral reasoning* is defined as a “conscious mental activity that consists of transforming given information about people in order to reach a moral judgment. To say that moral reasoning is a conscious process means that the process is intentional, effortful, and controllable and that the reasoner is aware that it is going on.”^47^

One of SIM’s most significant observations is that moral judgments are typically made on the basis of moral intuitions, which are then followed by slow, ex-post-facto moral reasoning.^48^ The classic phrase by the philosopher David Hume, “reason is, and ought only to be[,] the slave of the passions,” captures the fundamental idea underlying this observation.^49^ In other words, moral reasoning is a post-hoc rationalization and is rarely the direct cause of moral judgment; people typically begin by holding a particular intuition, and then they look for justifications that support that intuition. From this observation, Haidt draws the conclusion that individuals ultimately become lawyers “trying to build a case rather than” judges “searching for the truth.”^50^ As an example, someone may feel morally opposed to abortion and later rationalize that intuition by arguing that life begins at conception.

SIM, therefore, implies that moral reasoning is not always effective in motivating one’s moral judgments. Like other types of judgment, such as aesthetic judgment, moral judgment is instantaneous; people have an immediate sense of approval or disapproval. It is essential to emphasize that SIM does not deny the reasoning process; rather, it simply doubts the causality of reasoning in moral judgment (i.e., whether moral reasoning is the *cause*, rather than the *consequence*, of moral judgment). SIM acknowledges the complexity of generating moral judgments, recognizing that they result from intuition, reasoning, and social influences all together.

Haidt presents the following example to simplify SIM’s observation and make it more concrete:

[T]he President makes his decisions first and then dispatches the press-secretary to justify and rationalize those decisions. The press secretary may have no access to the real causes of the President’s decision, and is therefore free to make up whatever argument will sound most convincing to the general public. Everyone knows that it serves no purpose to argue with the press secretary. Convincing her that her arguments are specious or that the President’s decisions are wrong will have no effect on the president’s decisions, since those decisions were not based on the press secretary’s arguments.^51^

When applied to the context of moral experts in health policymaking, SIM’s observation suggests that experts’ moral judgment may not always be the outcome of deliberate reasoning and reflection. It posits that when confronted with moral dilemmas, moral experts, like any of us, may make moral judgments intuitively and reason about those judgments afterward. Though they may be motivated by their moral intuitions, moral experts may provide normative justifications in defense of their preexisting moral intuitions and judgments.

A second observation of SIM is that moral judgments are not fixed but are the product of one’s upbringing and social environment.^52^ SIM suggests that “moral reasoning is produced and *sent forth verbally* to justify one’s already-made moral judgment to others.”^53^ Haidt refers to this phenomenon as the “reasoned persuasion link.” He explains that this type of persuasion, which is composed of reasons and arguments, seems to have a causal effect: it can challenge the arguments of others and stimulate new intuitions in those who are listening.^54^ Haidt further points out that even in circumstances where no reasoned persuasion is offered, “the mere fact that friends, allies, and acquaintances have made a moral judgment exerts a direct influence on others.”^55^

Overall, this observation indicates that moral judgment is an ongoing interpersonal and social process — a person’s moral judgment is directly influenced by the moral judgment of individuals around them. Individuals rarely override their intuitive moral judgment just by arguing to themselves in private; thereby, moral reasoning only has a causal role in moral judgment when it runs through the minds of others.

It follows, then, that moral experts who consistently and continuously discuss moral questions with other individuals — starting during their training (where they discuss with other trainees and mentors) and continuing in their professional careers (where they discuss with colleagues, members of the public, and other stakeholders) — may have a more developed moral judgment as a result of running their moral intuitions and judgments by others. Through reasoned persuasion, it seems possible to bring reasoning into experts’ ordinarily intuitive moral judgments and get them to view a particular moral issue differently. This is especially useful due to the fact that moral experts may well lack sufficient input from their daily lives in society.

SIM’s second observation, I believe, signifies that bringing in diverse viewpoints to health policymaking and not demoting the voices of non-experts, including the voices of marginalized groups who are frequently ignored and with whom experts do not usually interact, could help shape experts’ moral intuitions and judgments in ways that better reflect the public’s interests and needs. By allowing different voices to be heard and considered in discussions related to moral questions, new intuitions can be developed, and experts (and other stakeholders like policymakers) can be pushed to see a particular moral problem from a new perspective. After all, as SIM interestingly suggests, when it comes to morality, people’s judgments rely heavily on the judgments of others around them.

By incorporating multiple viewpoints in health policymaking, it is possible to not only influence experts’ moral judgments, but also to achieve epistemic justice and add democratic legitimacy to policymaking processes.^56^ By epistemic justice, I mean a society that considers not only the distribution of its resources but also who has a voice and contributes knowledge.^57^ To achieve epistemic justice, we must build the capacity to comprehend the experiences of others and accord them the respect they deserve as sources of knowledge. For health policymaking processes to be fair, they must foster thorough public deliberation and democratic oversight.^58^ Experts’ moral judgments should not be seen as the sole authoritative source, nor should they be given more consideration than the moral judgments of those directly affected by the policy and other members of society.

It is beyond the scope of this article to delineate a decision-making model for health policymaking. I do wish to note, however, that an ideal decision-making model would not only involve listening to diverse viewpoints. Instead, it would incorporate a “co-design mechanism,” which involves different stakeholders and expertise and promotes *collaborative decision-making processes*.^59^ Namely, rather than having moral experts impose their vision and ideas as if they were the only alternative, a co-design mechanism would value all voices and provide the power to be actively involved in the design of health policies to a wide range of stakeholders, including provocateurs, individuals whose interests are directly affected, and members of the public.

A co-design mechanism is supported by transdisciplinary approaches to problem-solving and draws on established traditions of participation, collaboration, and empowerment in public policy. Transdisciplinary approaches to health policymaking include experts from multiple disciplines and fields, practitioners, policymakers, and members of the public “who together offer a broad array of relevant knowledge and points of view.”^60^ They improve health-related practices and policies due to the breadth of experiences they bring to the table. Likewise, different approaches to democratic governance enable a much larger group of stakeholders to be represented and foster a collective consciousness that binds the different stakeholders to one another.^61^

## Part III: Theory to Practice: Applying the Social Intuitionist Model in the Real World

In the previous part, I argued in favor of using SIM to guide health policymaking. I first claimed that the current expert governance structure does not account for the intuitive basis of moral judgments. I then suggested that we should stop taking moral experts’ policy recommendations at face value, and instead make it a priority to guarantee that different stakeholders have an equal chance to participate in health policymaking. Public engagement is a more promising path for democratic governance of health because it helps to better acquire reasoned and a community-based perspective. It is only when all points of view are fairly represented that the policy can be said to be legitimate.

When put into practice, SIM suggests that the “elitist” approach to health governance, which heavily relies on moral (and non-moral) experts, is one we ought to reject. Experts and other stakeholders should work toward revealing underlying moral proclivities, build working relationships, empower different individuals to raise their voices, explore different perspectives by listening, and collaborate rather than remain entrenched in their positions.

In addition, SIM suggests that it is of the utmost importance to offer possibilities for the public to engage in health policymaking. Historically, the public has been able to vote, and it may sometimes participate in institutional participatory processes (such as the notice‐and‐comment process). Yet, such opportunities by themselves are insufficient. There should be more ways for people to be involved in planning and designing health policies, and members of the public should be able to make their thoughts known in various ways.

So how would SIM be useful in real-world applications? This section uses the governance of gene-editing technologies as a case study to better understand SIM’s practical implications.

As a result of technological advancements in gene editing, a wide variety of organisms, including plants, animals, and even humans, are now capable of having precise genetic alterations applied to them.^62^ Gene-editing technologies have the potential, for instance, to generate crops that are more resistant to the effects of climate change;^63^ improve animal welfare and production;^64^ and enhance human health by removing disease-causing mutations.^65^ The idea of introducing heritable modifications into human embryos has thus far garnered the most interest. Many ethical, social, and legal questions have been raised about these emerging technologies, including whether they are safe, whether they should be used for non-therapeutic and enhancement purposes, what effect they will have on future generations, and whether they will exacerbate existing gaps in access to health.

The controversy reached a turning point in 2018 when a Chinese researcher, Dr. He Jiankui, claimed to have altered the genomes of twin baby girls to make them HIV immune.^66^ In the wake of He’s announcement, a number of initiatives were set up to foster discussion on the ethical, social, and legal questions raised by the use of gene-editing technologies on humans,^67^ including several international summits convened by the U.S. National Academy of Sciences and U.S. National Academy of Medicine, the Royal Society of the United Kingdom. These initiatives were dominated by expert voices.^68^

As several scholars have observed, when it comes to the governance of gene-editing technologies, experts have taken the reins and the debate “has moved… [to a model] enacted through high-level expert groups with little or no public input.”^69^ In many initiatives, “a cross-section of patients, affected communities, and the ‘general public’ were strikingly absent.”^70^ Scholars have highlighted that although some initiatives called for a “broad public dialogue,” they are ultimately “constrained by expert accounts of what is proper (and not proper) to talk about in ensuing deliberations.”^71^

The use of moral experts as sources of reason is a common practice in health policymaking. In this article, I have argued that health policymaking cannot be conducted in a truly reasoned environment in its current form. It seems that the “view from nowhere” approach is embraced, leading governments to rely on the moral judgment of moral experts and assume that these experts assess moral issues from an impersonal perspective.

Returning to SIM, I argue that choices regarding how to govern gene-editing technologies should not be decided solely by moral and non-moral experts. International dialogues should be held among a wide range of stakeholders, especially considering the potential global impact of these technologies. It has become evident, I hope, that merely holding expertise is not sufficient to guarantee that experts would make unbiased decisions based on reasoned judgment. What we truly need to restore the rationalism that is desired of policy related to gene-editing technologies (and health more broadly) is for the conversation to include a more diverse group of stakeholders. Inclusive deliberation is important not only to ensure that the voices of these stakeholders are considered, but also to ensure that experts internalize these voices and can be more productive participants in the conversation over the governance of gene-editing technologies.

As excellently expressed by Alessandro Blasimme, a senior researcher in bioethics at the Swiss Federal Institute of Technology:

[L]iberal democracies should ensure that dissent and disagreement can emerge anytime to challenge previously attained consensus. The value of including a plurality of views in democratic deliberation about controversial science is that it enables dissent and provides opportunities to frame what’s at stake. Expert committees can succeed in coordinating temporary solutions that avoid premature research or clinical applications. However, only inclusive deliberation can confer democratic legitimacy on decisions that can affect the future of humanity.^72^

It is worth noting that several interesting initiatives regarding the governance of gene-editing technologies appear to be promising. One initiative, the “Australian Citizens’ Jury on Genome Editing,” has been developed by a group of researchers.^73^ This initiative designed a mechanism for including community members in the conversation over human gene editing. Its primary objective has been to establish a forum in which ordinary citizens could advance their understanding of the ethical, legal, and social implications of human gene editing, investigate their positions on this technology, and collaborate to develop shared recommendations. This initiative served as the impetus for the formation of a larger initiative, which calls for the development of a global citizens’ assembly on genome editing that comprises more than one hundred individuals from all around the world.^74^

Other initiatives include the “Association for Responsible Research and Innovation in Genome Editing” and the “Global Observatory.” Both initiatives foster the kind of necessary public discussion and do not leave decisions like whether to modify genes, and if so, which ones, only to experts.^75^ These initiatives hope to provide “a comprehensive setting for all stakeholders (academics, private companies, patient organizations, citizens, decision makers) to allow the development of these paramount technologies in a safe and socially-acceptable environment”^76^ and to “convene communities that have not otherwise been in a position to reflect upon each others’ perspectives on issues that concern all humanity.”^77^

## Conclusion

The use of moral experts as sources of reason is a common practice in health policymaking. In this article, I have argued that health policymaking cannot be conducted in a truly reasoned environment in its current form. It seems that the “view from nowhere” approach is embraced, leading governments to rely on the moral judgment of moral experts and assume that these experts assess moral issues from an impersonal perspective.

As I have claimed, expert governance and its underlying “view from nowhere” approach are problematic for two main reasons. First, they fail to acknowledge that moral experts might not approach moral questions from a reasoned standpoint; they assume their moral judgment is free from distortions. However, as beautifully stated by Jonathan Haidt, “each of us is flawed as an individual reasoner.”^78^ Second, they presuppose that moral experts act impartially for the benefit of the public and disregard the necessity of critically evaluating experts’ moral judgment by other stakeholders. Therefore, there are substantial concerns that expert governance and the “view from nowhere” cannot adequately represent the interests and needs of the public.

We have reached a point where the formulation of health policy demands a comprehensive rethinking rather than a superficial or piecemeal approach to reform. The future of health should involve the development of new forms of national and international collaborations to create solutions to health challenges. In this article, I have asserted that health policymaking ought to involve active listening, education, and genuine conversation in order to foster alternative viewpoints and result in better health outcomes. If we are genuinely devoted to democratic decision-making processes in the health sphere, we must look at health-related moral questions from multiple perspectives before rendering moral judgments and we should encourage a diverse set of stakeholders to co-create health policy. I believe that approaching difficult moral questions in this manner is the most fruitful course of action.

## Note

The author has no conflicts to disclose.
